# Case Report: A Case of X-Linked Agammaglobulinemia With High Serum IgE Levels and Allergic Rhinitis

**DOI:** 10.3389/fimmu.2020.582376

**Published:** 2020-11-05

**Authors:** Bianca Cinicola, Andrea Uva, Lucia Leonardi, Daniele Moratto, Silvia Giliani, Rita Carsetti, Simona Ferrari, Anna Maria Zicari, Marzia Duse

**Affiliations:** ^1^ Department of Pediatrics, Policlinico Umberto I, Sapienza University of Rome, Rome, Italy; ^2^ Cytogenetic and Medical Genetics Unit, “A. Nocivelli” Institute for Molecular Medicine Spedali Civili Hospital, Brescia, Italy; ^3^ Flow Cytometry Unit, Clinical Chemistry Laboratory, Spedali Civili Hospital, Brescia, Italy; ^4^ Department of Molecular and Translational Medicine, University of Brescia, Brescia, Italy; ^5^ B Cell Physiopathology Unit, Immunology Research Area, Bambino Gesù Children Hospital, Rome, Italy; ^6^ Medical Genetics Unit, S.Orsola-Malpighi University Hospital, Bologna, Italy

**Keywords:** X-linked Agammaglobulinemia, *BTK* gene, protein expression, mild phenotype, IgE production, allergy

## Abstract

X-linked Agammaglobulinemia (XLA) is a rare genetic disorder of B-lymphocyte differentiation, characterized by the absence or paucity of circulating B cells, markedly reduced levels of all serum immunoglobulin isotypes and lack of specific antibody production. Bruton Tyrosine Kinase (*BTK*) gene encodes a cytoplasmic tyrosine kinase involved in the B cell maturation and its mutation, blocking B cell differentiation at the pre-B cell stage, and is responsible for XLA. All domains may be affected by the mutation, and the many genotypes are associated with a wide range of clinical presentations. Little is known about genotype-phenotype correlation in this disorder, and factors influencing the phenotype of XLA are not clearly understood. In this report we present a unique case of a young patient affected by XLA. The disease was genetically diagnosed at birth due to a family history of XLA, but during follow up, it was characterized by a CD19+ B cell percentage consistently greater than 2%. He never suffered severe infections, but at two years of age, he developed persistent rhinitis. Thus, total serum IgE levels were measured and detected over the normal range, and specific allergic investigations showed sensitization to dust mites. Further immunological tests (BTK expression, functional “*in vitro*” B cell proliferation upon CpG stimulation, B cell subset analysis) explained these findings as possible manifestations of a mild XLA phenotype. XLA patients rarely present with allergic manifestations, which could warrant further investigation. High serum IgE levels could be a sign of a mild phenotype, but their role and the mechanisms underlying their production in XLA need to be clarified.

## Introduction

X-linked Agammaglobulinemia (XLA) is a primary immunodeficiency disease caused by a mutation in the Bruton Tyrosine Kinase (*BTK*) gene that encodes an essential protein involved in B-cell maturation. In particular, it promotes preB cell expansion at the preB1 to preB2 stage (1, 2). The *BTK* gene is located in the Xq22 region of the X chromosome and has five distinct structural domains: Pleckstrin homology (PH), Tec homology (TH), Src homology (SH3), SH2, and catalytic kinase (SH1) domains. All domains may be affected by mutations. Among all the XLA causative mutations, 15% to 20% are known to occur *de novo* (3). Carrier mothers are usually healthy, even if there is evidence of XLA syndrome in females due to skewed X-chromosome inactivation (4).

Failure of B-cell development caused by a *BTK* gene mutation in affected males leads to reduced levels of peripheral mature B lymphocytes, plasma cells and all immunoglobulin (Ig) isotypes (5). As a consequence, XLA patients are more susceptible to recurrent respiratory infections, mostly due to encapsulated pyogenic bacteria, and bowel infections caused by Salmonella, Yersinia, Campylobacter and Giardia (6). Therapy mostly consists of Ig replacement, with intravenous (IVIG), subcutaneous (SCIG), or enzyme facilitated immunoglobulin (7).

A definitive diagnosis of XLA is possible, based on the European Society for Immunodeficiencies (ESID) criteria, in a male presenting with CD19^+^ B cells < 2% and a confirmed mutation in the *BTK* gene and/or an absent BTK protein expression, and/or a male family member of maternal lineage with CD19+ B cells <2% (8).

Despite defined diagnostic criteria, a wide number of BTK mutations is associated with different clinical phenotypes, including several atypical or leaky XLA forms (9–11).

Different mutations in the *BTK* gene, classified according to their severity by Conley and Howard (12), may influence the severity of the disease (13) and result in a considerable heterogeneity in the clinical spectrum of XLA (14); however, genotype-phenotype correlation has not been clearly established (15).

Allergy can also occur in XLA patients. Both IgE mediated and non-IgE mediated symptoms have been reported. In particular, Melo et al. (16) described a patient with XLA and non IgE mediated cow milk protein allergy. Besides, IgE mediated allergy have been reported in two different studies (17, 18) describing two XLA patients with a severe course of disease, low serum immunoglobulin isotypes except for normal IgE levels, and hypersensitivity to several allergens. Recently, another study (19) identified an atypical case of XLA diagnosed at the age of 45, characterized by CD19+ B cells 1%, mild hypogammaglobulinemia and detection of serum IgE and allergen-specific IgE for cedar pollen and alternaria.

Finally, Kaneko et al. (20) reported high serum IgE levels in a mild XLA patient presenting with sensitization to dust mites and partial BTK expression on lymphocytes. Interesting, the authors assumed IgE levels as a critical marker for the detection of the leaky phenotype.

However, to the best of our knowledge, none of the studies on allergic XLA patients reported detailed immunological assessment.

Our study describes a unique case of a patient affected by XLA characterized by CD19+ B cells > 2% and allergic disease, with high serum IgE levels, persistent rhinitis and sensitization to dust mites.

Moreover, we performed a complete B cells subset analysis and a functional test on B lymphocytes along with BTK expression measurement, in order to better characterize his phenotype.

## Case Presentation

We report the case of a 10-year-old boy admitted to our primary immunodeficiency disease (PID) outpatient service at three months of age with a diagnosis of XLA. The molecular test was performed at birth due to a positive family history (maternal uncle), identifying the presence of the *BTK* mutation (c.82C>T:p.Arg28Cys).

In the first three months of life, the patient was in a good physical condition; he never suffered severe infections and his growth was at the 75^th^ percentile. His first blood test showed a normal white blood cell (WBC) count (5.400 lymphocytes/mm^3^), a CD19+ B cell percentage of 6% and reduced serum Ig levels: IgG 430 mg/dl (presumably mostly due to trans placental passage), IgA <1 mg/dl, IgM <1 mg/dl and IgE 4 mg/dl. Immunoglobulin replacement (400 mg/kg every 21 days) was started one month later to prevent severe infections. At two years of age, he converted to subcutaneous immunoglobulin therapy (SCIG) and, from the age of eight, he started hyaluronidase-facilitated SCIG.

Although the patient has never presented with severe infections, he developed persistent rhinitis at two years of age, successfully treated with antihistamines and topical corticosteroid. Despite the deficit of Ig production, total serum IgE levels were above the normal range (246 mg/dl), with normal levels of eosinophils in peripheral blood. Subsequently, serum IgE levels were persistently elevated with a maximum of 936 mg/dl at the age of eight, and were always associated with normal eosinophil counts. Considering a possible atopic condition, skin prick test (SPT) was performed highlighting sensitization to dust mites, confirmed by allergen-specific IgE levels to *dermatophagoides pteronyssinus* (DPT) (IgE=2,44 kU/L).

**Figure 1:**
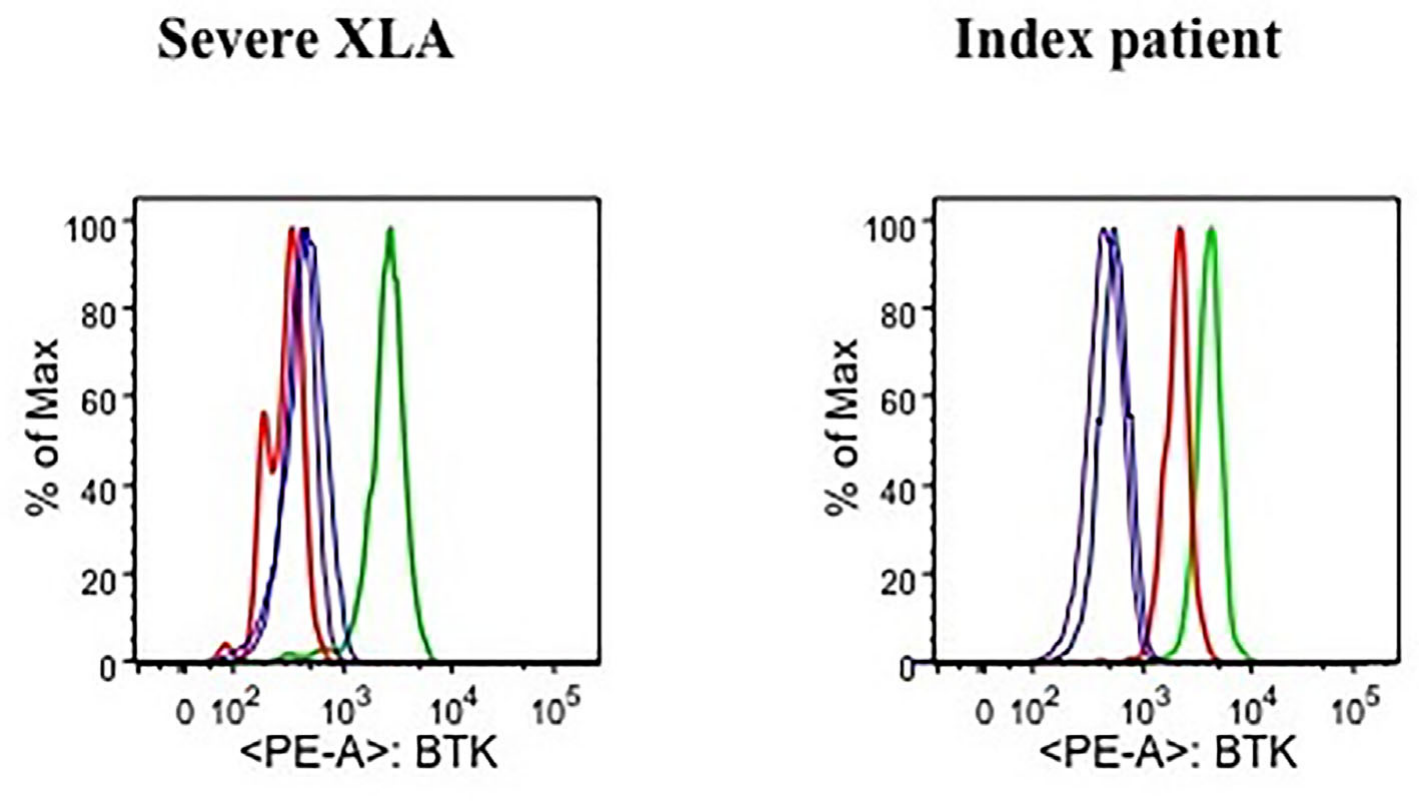
Flow cytometric analysis of BTK expression in a severe XLA patient (left plot) and the index patient (right plot). For each plot, histograms representing BTK expression measured as Mean Fluorescence Intensity (MFI) are presented for the following cell subsets: B lymphocytes of a healthy control (green line), patient’s B lymphocytes (red line), T lymphocytes of a healthy control (blue line), patient’s T lymphocytes (purple line).

Evaluation of lymphocytes over the years demonstrated a CD19+B cell percentage consistently greater than 2% (range, 2–7%) with absolute counts of at least 43 cells/μl. Functional “*in vitro*” analysis of B lymphocytes, upon response to CpG stimulation, showed a mild proliferative ability and impaired plasma cell differentiation coupled with the absence of IgA class antibodies in the supernatants, but detectable IgM and IgG levels. To determine if clinical and laboratory findings of the patient (including elevated total IgE serum levels) could be explained by a mild XLA phenotype, BTK expression was measured by an indirect flow cytometric test performed after staining blood cells with a commercial anti-BTK monoclonal antibody (Becton Dickinson), followed by incubation with a secondary PE-conjugated antibody (southern Biotech). Flow cytometry analysis displayed the presence of a residual BTK protein expression of both patient’s B lymphocytes (**Figure 1**) and myeloid lineage cells (monocytes and granulocytes). To note, BTK was identified as a single peak for each population, with a Mean Fluorescence Intensity (MFI) corresponding respectively to approximately 40% (**Figure 1**) and 70% (data not shown) of the values measured on the healthy control cells. Conversely, BTK was absent in circulating B lymphocytes of a patient diagnosed with a severe form of XLA, whose MFI was comparable to one of those lymphocyte subsets which intrinsically lack BTK expression, such as T cells (**Figure 1**).

**Figure 2:**
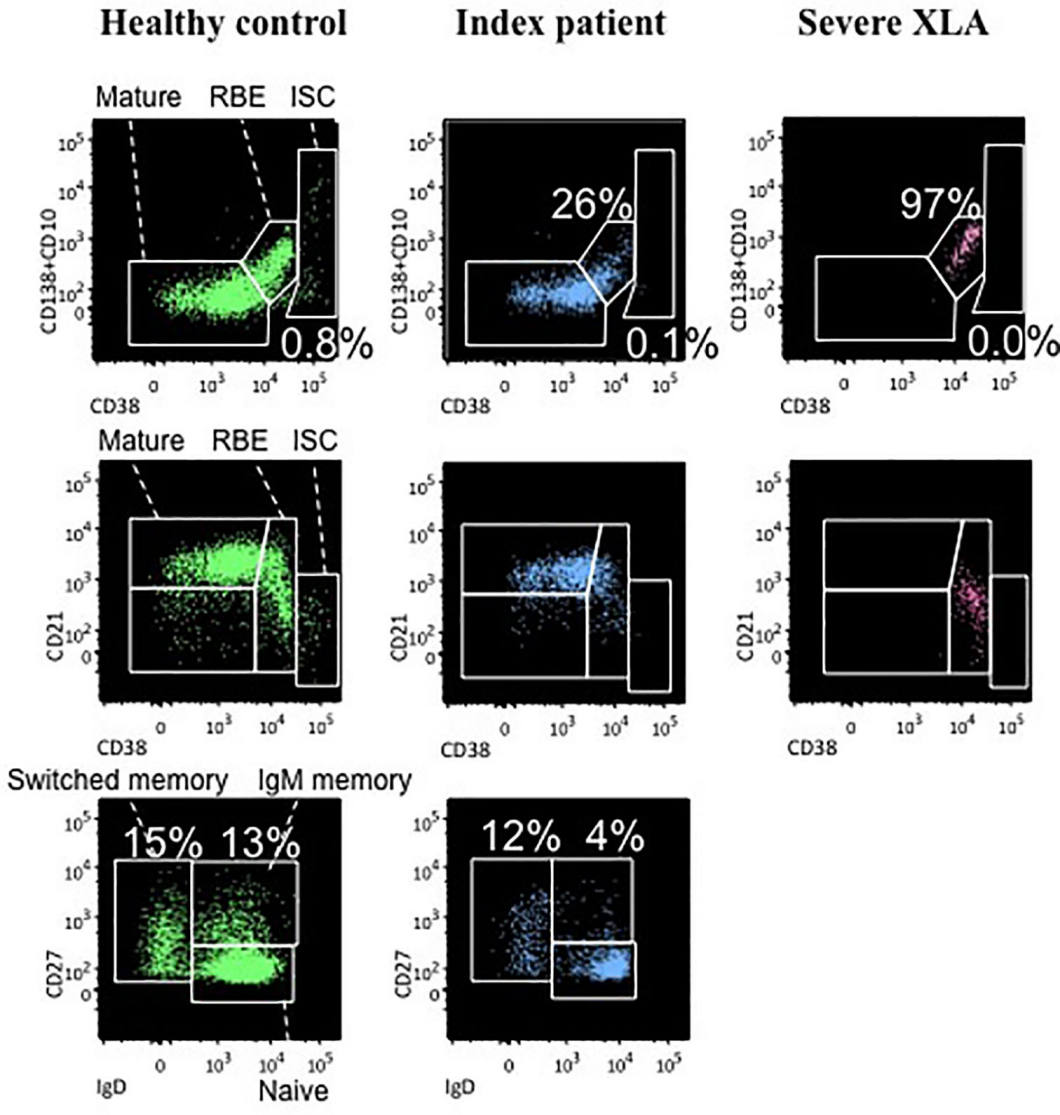
Analysis of peripheral B cell subset distribution a healthy control (left column), the index patient (central column), and a severe XLA patient (right column). For each subject the following B cell subsets are presented: recent bone marrow emigrants (RBE) (CD20+CD38++CD10+), immunoglobulin-secreting cells (ISC) (CD38+++CD27++CD20-) and mature cells (CD20+CD38+/-CD10-) which are further classified in naïve cells (IgD+IgM+CD27-) and both switched memory (IgD-IgM-CD27+) and unswitched memory cells (IgD+IgM+CD27+).

Evaluation of peripheral B lymphocytes by flow cytometry demonstrated the presence of a normal distribution of CD20+ cell subsets. Percentages of cells having the phenotypical characteristics of recent bone marrow emigrants (RBE) (CD20+CD38++CD10+) and naïve cells (IgD+IgM+CD27-), as well as switched and unswitched memory cells (IgD-IgM-CD27+ and IgD+IgM+CD27+ respectively), did not significantly differ from values observed in healthy age-matched individuals, while only immunoglobulin-secreting cells (ISC) (CD38+++CD27++CD20-) were below the normal range (**Figure 2**, central column). On the opposite, peripheral B cells of XLA patients with severe clinical manifestations and undetectable BTK expression did not display any ability to differentiate into mature cells (**Figure 2**, right column).

## Discussion

*BTK* gene mutations are collected in the BTKbase, which is an XLA-mutation registry established in 1994 (21). This database reports the distribution as well as the frequency of the different mutations in the protein domains.

So far, a total of 620 mutations in 974 unrelated families have been recorded (22), with heterogeneous XLA clinical presentations, which are often associated to the same mutation, making a genotype-phenotype correlation challenging to define among XLA patients, even in studies including a high number of patients (23). However, some studies showed that mild mutations, defined as mutations that may allow a residual protein function, could be linked to late-onset of disease, lower number of hospitalizations and severe infections before diagnosis, as well as higher Ig levels at diagnosis (24–27). These studies included patients carrying a missense mutation in the PH domain that involved the R28C residue (**Table 1**) and presented with a milder disease despite very low circulating B cells (lower than 2%) and low serum Ig levels. On the contrary, another study described a patient with the same amino acid substitution as our patient, but with a severe clinical phenotype characterized by several infections since 12 months of age (28). Thus, it is quite difficult to predict the clinical evolution of XLA patients based only on *BTK* gene analysis. It is possible that other factors, including epigenetic variables, could influence the course of the disease (29).

**Table 1:** Immunologic features and BTK expression in XLA patients with R28C substitution reported in literature.

Author	Nucleotide Change	Amino Acid Substitution	IgG (mg/dl)	IgA (mg/dl)	IgM (mg/dl)	CD19 (%)	BTK Protein	Phenotype
(24)	46278C>T	R28C	410	53	16	1	Expressed	Mild
(25)	NA	R28C	NA	NA	NA	NA	Expressed	Mild
(26)	214C>T	R28C	267	<0.1	<0.1	0.1	NP	Mild
(27)	214C>T	R28C	267	<10	<10	0.1	NP	Mild
(28)	214C>T	R28C	30	5	5	0.1	NP	Severe

NP not performed NA not reported.

However, *BTK* gene analysis combined with study of protein expression might help in predicting the clinical phenotypes. Therefore, to better elucidate the genotype-phenotype correlation, two of the previously described studies analyzed BTK expression, which was found to be positive in some patients with amino acid substitutions, including R28C. At the same time, other mild mutations resulted in the absence of BTK protein in the cells analysed (24, 25). These results suggest that a milder disease could be explained by some residual function of the mutated protein, but the correlation between genotype, phenotype and protein expression is not clear yet, and further studies are needed to clarify this aspect.

Our patient displayed typical clinical and immunophenotypical characteristics of subjects harboring permissive mutations. Number and severity of clinical manifestations correlate with the presence of three different leukocyte subsets expressing a reduced amount of BTK protein, as measured as a single fluorescence peak in a flow cytometric test. These are characteristics compatible with a mutation with residual protein expression but not with the presence of cells carrying one or more reversions of the original mutation. In fact, although revertant cells are not so uncommon in primary immunodeficiency diseases (30), they are limited to highly differentiated cells of those leukocyte subsets for which the reversion confers the most selective advantage (Moratto D. and Giliani S. unpublished data on Wiskott-Aldrich Syndrome and X-linked severe combined immunodeficiency revertant patients). Moreover, the high percentages of RBE and naïve cells are also in contrast with the hypothesis of a selection of revertant clones, and suggest the presence of B cells with a polyclonal repertoire, although a specific test to evaluate the clonality of the index patient’s B cells was not performed.

In contrast to our findings, none of the above mentioned studies reported information on serum IgE levels and allergic symptoms in the XLA patients described. Indeed, typical XLA patients have no serum IgE. To the best of our knowledge, among XLA patients with R28C substitution, our patient seems to be the first subject with high serum IgE levels and sensitization to dust mites. Kaneko et al. (20) assumed that elevated IgE levels could be the critical marker in diagnosing mild XLA. They reported a patient carrying a different mutation (IVS11 + 3G>T mutation on SH2 domain), leading to 80-bp truncated band and normal size *BTK* gene transcripts. This patient had detectable CD19+B cells (2.1%), measurable IgG, IgM and IgA, elevated IgE levels (269 IU/ml) and allergen-specific IgE for house dust and mites. B cells could then proliferate due to antigen stimulation and differentiate into memory B cells, as in the case of our patient. Moreover, 31% of CD19+ B cells (but not monocytes) expressed normal BTK protein.

The reason for high IgE serum levels is still not clearly understood. Two studies drive hypothesis on the IgE production in XLA patients.

Nonoyama et al. (31) analyzed four XLA patients with missense mutations, and one without a genetic diagnosis, characterized by circulating CD19+B cell levels < 1% and lack of BTK protein expression. After stimulation with anti-CD40 and IL-4, B-lymphocytes proliferated and produced quantities of IgE almost comparable to the controls (patient IgE not detectable prior to stimulation). This study speculates that “leaky” B cells from XLA patients could differentiate using *BTK*-independent pathways, such as a CD40-controlled pathway.

Another study (32) reported the case of a 7-year-old boy with XLA (Del E407 in the TH domain), who presented at diagnosis with CD19+B cells <1%, low expression (0.70%) of BTK in monocytes, and low Ig levels of all isotypes, including IgE, but a positive sensitization to various inhalant allergens and allergic symptoms since the age of one. The authors supposed bias towards a T helper 2 pattern, since cytokines secreted by T lymphocytes in response to allergic stimuli can determine induction and maintenance of allergic inflammation, but further studies on the T cells of XLA patients are required to establish the pathophysiology of that process.

In conclusion, X-linked Agammaglobulinemia is an immune disorder that manifests as different clinical phenotypes, from severe to a milder course of the disease. Much of the debate has been focused on the possible relationship between mutations in the *BTK* gene and clinical phenotypes, with inconclusive results. As high serum IgE levels have been detected in XLA patients and their role and importance are still in the spotlight, all patients with mild mutations should be studied further in order to clarify the role of IgE production in influencing the severity of the disease and its prognostic significance.

## Data Availability Statement

The raw data supporting the conclusions of this article will be made available by the authors, without undue reservation.

## Ethics Statement

Written informed consent was obtained from the minor’s legal guardian for the publication of any potentially identifiable images or data included in this article.

## Author Contributions

All people who meet authorship ICMJE criteria are listed as authors, and all authors certify that they have participated equally in the work to take public responsibility for the content, including participation in the concept, design, analysis, writing, or revision of the manuscript. Furthermore each author certifies that this material or similar material has not been and will not be submitted to or published in any other publication. All authors contributed to the article and approved the submitted version.

## Conflict of Interest

The authors declare that the research was conducted in the absence of any commercial or financial relationships that could be construed as a potential conflict of interest.
